# Sensitive Quantification of Cell-Free Tumor DNA for Early Detection of Recurrence in Colorectal Cancer

**DOI:** 10.3389/fgene.2021.811291

**Published:** 2022-01-05

**Authors:** Sebastian Stasik, Marika Mende, Caroline Schuster, Sandra Mahler, Daniela Aust, Andrea Tannapfel, Anke Reinacher-Schick, Gustavo Baretton, Claudia Krippendorf, Martin Bornhäuser, Gerhard Ehninger, Gunnar Folprecht, Christian Thiede

**Affiliations:** ^1^ Medical Department I, University Hospital Carl Gustav Carus, Technische Universität, Dresden, Germany; ^2^ National Center for Tumor Diseases (NCT), Partner Site Dresden, Heidelberg, Germany; ^3^ AgenDix GmbH, Dresden, Germany; ^4^ Institute of Pathology, University Hospital Carl Gustav Carus, Technische Universität, Dresden, Germany; ^5^ Institute of Pathology, Ruhr University, Bochum, Germany; ^6^ Department of Hematology, Oncology and Palliative Care, St. Josef Hospital, Ruhr University, Bochum, Germany

**Keywords:** cell-free DNA, cell-free tumor DNA, liquid biopsy, next-generation sequencing, colorectal cancer, persistence and recurrence

## Abstract

The detection of plasma cell–free tumor DNA (ctDNA) is prognostic in colorectal cancer (CRC) and has potential for early prediction of disease recurrence. In clinical routine, ctDNA-based diagnostics are limited by the low concentration of ctDNA and error rates of standard next-generation sequencing (NGS) approaches. We evaluated the potential to increase the stability and yield of plasma cell–free DNA (cfDNA) for routine diagnostic purposes using different blood collection tubes and various manual or automated cfDNA extraction protocols. Sensitivity for low-level ctDNA was measured in *KRAS*-mutant cfDNA using an error-reduced NGS procedure. To test the applicability of rapid evaluation of ctDNA persistence in clinical routine, we prospectively analyzed postoperative samples of 67 CRC (stage II) patients. ctDNA detection was linear between 0.0045 and 45%, with high sensitivity (94%) and specificity (100%) for mutations at 0.1% VAF. The stability and yield of cfDNA were superior when using Streck BCT tubes and a protocol by Zymo Research. Sensitivity for ctDNA increased 1.5-fold by the integration of variant reads from triplicate PCRs and with PCR template concentration. In clinical samples, ctDNA persistence was found in ∼9% of samples, drawn 2 weeks after surgery. Moreover, in a retrospective analysis of 14 CRC patients with relapse during adjuvant therapy, we successfully detected ctDNA (median 0.38% VAF; range 0.18–5.04% VAF) in 92.85% of patients significantly prior (median 112 days) to imaging-based surveillance. Using optimized pre-analytical conditions, the detection of postoperative ctDNA is feasible with excellent sensitivity and allows the prediction of CRC recurrence in routine oncology testing.

## 1 Introduction

Cell-free DNA (cfDNA) represents a fraction of molecules that are constantly released into the blood circulation and other body fluids by cellular processes of necrosis or apoptosis (Schwarzenbach et al., 2011). In malignant diseases such as cancer, plasma cfDNA is enriched with circulating cell-free tumor DNA (ctDNA) and may carry tumor-derived genetic and epigenetic aberrations, reflecting the clonal architecture and evolution of corresponding cancer cells in the primary tumor tissue (Schwarzenbach et al., 2011; Crowley et al., 2013). Various molecular tumor-specific alterations have been detected in ctDNA, including DNA hypermethylation and point mutations in relevant genes such as *KRAS* and *TP53* (Strickler et al., 2018; Luo et al., 2020).

As the release of ctDNA from tumor cells is related to the state and size of the tumor, levels of ctDNA may vary owing to cancer development and progression (Schwarzenbach et al., 2011; Crowley et al., 2013; Reinert et al., 2016). Consequently, the detection of ctDNA in plasma has potential to serve as a highly specific and low-invasive “liquid biopsy” for the early prediction of disease recurrence in clinical routine (Tie et al., 2016; Wan et al., 2017). The importance of ctDNA detection as prognostic biomarker has already been demonstrated in a variety of malignancies, including breast, lung, bladder, colon, and pancreatic cancer (Bettegowda et al., 2014). Due to its relevance as the second leading cause of cancer-related deaths and improvements in targeted therapies, plasma ctDNA has also been evaluated in colorectal cancer (CRC) with respect to risk stratification (Tie et al., 2016; Luo et al., 2020), prognosis (El Messaoudi et al., 2016; Wang et al., 2019), and prediction of treatment response or progression during postoperative surveillance (Siravegna et al., 2015; Tie et al., 2015; Reinert et al., 2016; Tie et al., 2016).

Although the presence of ctDNA is indicative for residual disease, the sensitive detection of ctDNA as early predictive marker is typically limited by the low frequency of mutant alleles in peripheral circulation (e.g., <0.1%) and the low amount of available cfDNA as PCR template, respectively (Wan et al., 2017). Several next-generation sequencing (NGS) approaches potentially enable the quantification of mutant alleles at ultra-deep frequencies but often require high amounts of template cfDNA, complex molecular barcoding strategies, and/or extensive bioinformatics, which impairs rapid translation into clinical practice (Kastrisiou et al., 2019). Furthermore, the quality and quantity of cfDNA as laboratory analyte is substantially affected by the short half-life time (typically 15 min–3 h) and potential contamination with genomic DNA (gDNA) from white blood cells during the pre-analytical phase of blood collection (Medina Diaz et al., 2016; Wan et al., 2017).

In order to improve processing of plasma cfDNA for diagnostic purposes, we evaluated the potential to increase stability and yield of cfDNA for NGS downstream applications using adequate blood collection tubes (Streck, EDTA tubes) as well as various manual (Zymo Research, Qiagen, Analytik Jena) and automated (QIAsymphony) cfDNA extraction procedures. To come up with a rapid and robust method for routine laboratory testing, we performed a comprehensive evaluation of ctDNA detection (variable template concentrations and integration of variant reads from multiple PCR replicates) at variant allele frequencies in a clinically relevant range of 0.01–0.1%, using an optimized error-reduced deep sequencing procedure (Stasik et al., 2018). To test the feasibility of our approach for early prediction of disease recurrence in clinical settings, 104 serial plasma samples of 14 patients with advanced CRC (stages II-IV) and relapse during adjuvant therapy post-tumor resection were retrospectively analyzed for the detection of known hot spot mutations (*KRAS*, *NRAS*, and *TP53*) in cfDNA and compared to imaging-based surveillance. In addition, we document the applicability of this approach for rapid prospective evaluation of ctDNA persistence in 67 patients diagnosed with stage II CRC.

## 2 Materials and Methods

### 2.1 Patient Samples

All samples and clinical data were obtained with written informed consent of the patients. All studies involving human primary materials were performed after approval of the Local Ethical Board of the University Hospital Dresden and were in agreement with the Helsinki Declaration. For the analysis of postoperative ctDNA persistence (stage II CRC), patients were screened as part of the ColoPredict platform (AIO-KRK-0413) and subsequently registered for the ongoing prospective CIRCULATE-trial (NCT#04089631).

### 2.2 Blood Collection, Storage, and Plasma Preparation

To optimize ctDNA preservation during blood collection, blood from a *KRAS*-mutant CRC patient, with a c.38G > A variant allele frequency (VAF) of 35% (measured in peripheral blood by targeted sequencing), was diluted (1:1.75) in blood from healthy donors to obtain c.38G > A (p.Gly13Asp) VAFs of ∼20%. Whole blood mixtures were aliquoted using different blood collection tubes: conventional S-Monovette EDTA tubes (Sarstedt, Germany) and Cell-Free DNA BCT tubes (Streck, Omaha, NE, United States) containing a special buffer for stabilization of nucleated blood cells. In order to monitor ctDNA stability, collection tubes (4 replicates x 7 ml blood per tube) were subsequently incubated at room temperature (22°C) for a period of up to 14 days and processed for cfDNA extraction at 0, 24, 48, 72, 96, and 336 h post-collection. For separation of plasma, the blood samples were centrifuged at 300 g for 20 min. Without disturbing the buffy coat, the plasma layer (supernatant) was carefully removed and transferred into a new 2-ml low-bind tube. To completely remove residual cells, plasma samples were re-centrifuged at 5,000 g for 10 min, and the supernatant transferred to a new 2-ml low-bind tube and stored at −20°C until cfDNA extraction.

### 2.3 Extraction and Quantification of cfDNA

To evaluate potential impacts of cfDNA extraction procedures on total extraction yield and the detection of mutant alleles, plasma samples of a *KRAS* c.38G > A (10% VAF) positive CRC patient were processed using different manual protocols from various vendors: A Jena PME free-circulating DNA extraction kit (spin-based, carrier RNA: optional) (Analytik Jena, Jena, Germany), QIAamp Circulating NA Kit (vacuum-based, carrier RNA: yes) (Qiagen, Hilden, Germany), and Zymo Quick cfDNA serum and plasma kit (spin-based, carrier RNA: no) (Zymo Research, Irvine, CA, United States). In addition, cfDNA of matched plasma samples from CRC patients (*n* = 15) was extracted in parallel using the manual protocol by Zymo Research and an automated procedure on a QIAsymphony instrument (Qiagen) using the PAXcircDNA_STA_2400 protocol (Qiagen). All extractions were performed using 1–3 ml of plasma according to the manufacturer’s protocols. For all kits, cfDNA was eluted into 60 μL ddH2O (or TE buffer), quantified by a β-globin–specific qPCR in comparison to a serial dilution of a reference DNA with a known quantity on a 7,500 Real-Time PCR System (Applied Biosystems, Foster City, CA, United States), and stored at −20°C until downstream processing for NGS. All cfDNA concentrations are presented in ng mL^−1^ plasma to adjust for different volumes of starting material.

### 2.4 PCR Amplification and Deep Sequencing

Sequencing of cfDNA samples was performed according to an optimized protocol for error-reduced NGS-based detection of low-level single-nucleotide variants (SNVs) on an Ion Torrent instrument, as described previously (Stasik et al., 2018). Briefly, Fusion PCR primers for the preparation of amplicon libraries were designed (Primer Premier 6; Premier Biosoft, Palo Alto, CA, United States) according to the manufacturer’s recommendations (Fusion Method; Life Technologies). PCR on plasma cfDNA (40 cycles) was performed using the Q5^®^ High-Fidelity proofreading polymerases (New England Biolabs, Beverly, MA, United States). PCR primer sequences and specific PCR conditions for all target regions are listed in Supplementary Table S1. All PCRs were performed on a GeneAmp PCR System 9700 (Applied Biosystems, Foster City, CA, United States). After a two‐round purification process with Agencourt AMPure XP Reagent (Beckman Coulter, Krefeld, Germany), barcoded PCR products were quantified with a Qubit 2.0 fluorometer (Life Technologies) using the Qubit dsDNA HS Assay (Life Technologies). For deep sequencing, 25 µL of the diluted library (30 pM) was loaded on an Ion Chef instrument (Life Technologies) for automatic template preparation and sequenced unidirectionally on an Ion S5 XL NGS system (Life Technologies), according to manufacturer’s protocols and aiming at a ≥100.000-fold coverage per target region. Sequence data alignment of demultiplexed FastQ files, variant calling, and filtering was done using the Sequence Pilot software package (JSI Medical Systems GmbH, Ettenheim, Germany) with default settings. Human genome build HG19 (http://genome.ucsc.edu/) was used as reference genome for mapping algorithms. According to false-positive rates of individual targets (Supplementary Table S2), NGS-based ctDNA detection was conducted with a defined cutoff of 0.01%. VAFs below the predefined thresholds were considered ctDNA wild type (wt). Differences were analyzed using a two-sided Student t-test or the non-parametric Mann–Whitney *U* test. A p value of <0.05 was considered significant. All calculations were conducted using Prism 5 (GraphPad, La Jolla, CA, United States) and SPSS Statistics 25 (IBM, Armonk, NY, United States).

### 2.5 NGS Sensitivity for Low-Level ctDNA

To quantify the linear range of NGS-based ctDNA detection, cfDNA from a *KRAS* c.35G > A mutant patient sample (∼45% VAF) was serially diluted (10-fold) in wild-type (wt) cfDNA to obtain c.35G > A VAFs in the range of 0.0045–45%. NGS sensitivity for low-level ctDNA was measured in *KRAS* c.35G > A mutant cfDNA samples (*n* = 25) diluted to VAFs of 0.1%. To address the impact of available cfDNA as PCR template for the detection of mutant alleles, 5 or 20 ng of cfDNA was used for PCR amplification. The control cfDNA (*n* = 9) from healthy individuals (<50 years of age) served to assess per-base substitution error rates and specificity for the *KRAS* c.35G > A variant. To evaluate the potential benefit of integrating variant reads from multiple PCR replicates on NGS sensitivity, sequencing of 10 patient samples with the *KRAS* c.35G > A variant, diluted to 0.01% VAF, was performed using a pool of barcoded amplicons from triplicate PCR replicates (5 ng template each reaction) for parallel sequencing. The detection of ctDNA was compared to the analysis of corresponding samples using a single PCR with 20 ng of cfDNA as template.

### 2.6 Clinical Validation of ctDNA-Based Detection of CRC Persistence and Recurrence

In order to test the applicability of our approach for early detection of CRC recurrence in clinical settings, 104 serial plasma samples of patients (*n* = 14) with advanced CRC (stages II–IV) and relapse during adjuvant therapy were retrospectively analyzed for the detection of ctDNA. Time to detection of recurrence was compared between the detection of ctDNA and imaging-based surveillance. Furthermore, to evaluate the usability of ctDNA for rapid postoperative detection of CRC persistence, a prospective cohort of 67 patients with CRC stage II was screened for the detection of ctDNA in blood samples drawn ∼2 weeks after complete resection of all evident tumor tissue. For all clinical samples, tumor-specific mutations were determined in a formalin-fixed paraffin-embedded (FFPE) tumor material by NGS panel analysis in a central pathological laboratory. For each patient, 1–3 tumor-informed mutations were selected for ctDNA monitoring. Processing of cfDNA was performed according to optimized conditions, which were initially determined in the methodical evaluations. Briefly, blood (∼15 ml) was stored in Cell-Free DNA BCT (Streck) or PAXgene Blood ccfDNA tubes (Qiagen) and processed for plasma extraction within 7 days. cfDNA was extracted using the Zymo Quick cfDNA serum and plasma kit or the QIAsymphony instrument. PCR on individual samples was performed in triplicate using ≥5 ng of cfDNA (40 cycles) and the Q5 proofreading polymerase. Pooled libraries were sequenced as described earlier.

## 3 Results

### 3.1 Optimizing Blood Collection for Stability of ctDNA

**FIGURE 1:**
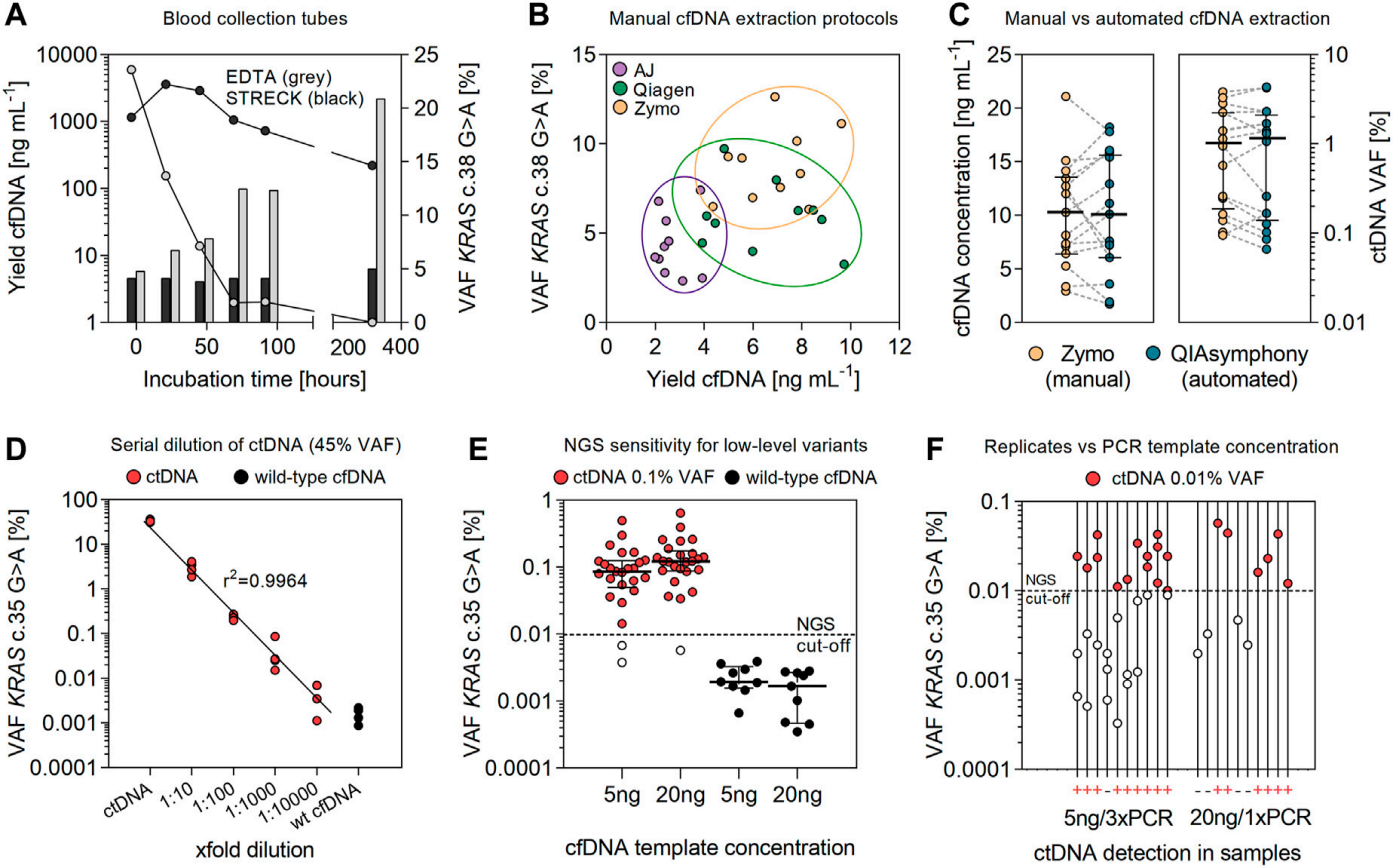
Optimizing cfDNA preservation and extraction for sensitive detection of ctDNA. **(A)** Stability of ctDNA in EDTA and Streck blood collection tubes. Data for plasma levels of cfDNA [ng mL^−1^ plasma] and the frequency of corresponding *KRAS* c.38G > A mutant alleles [%] represent mean values of multiple replicates (*n* = 4). **(B)** Comparing the efficiency of manual cfDNA extraction kits for total extraction yield [ng mL^−1^ plasma] and the detection of ctDNA [%]. Data are shown for different vendors: Analytik Jena (AJ), Qiagen, and Zymo Research. Extractions were performed in duplicate from plasma samples (1–3 ml) of CRC patients (*n* = 5). **(C)** Extraction yield [ng mL^−1^ plasma] and concentrations of detected ctDNA [%] in plasma samples (2.5 ml) of CRC patients (*n* = 15) (median coverage 121,825; range 70,027–179,931 reads). Extraction of cfDNA was performed using the manual protocol by Zymo Research and on a QIAsymphony instrument. Error bars represent median values and interquartile range. **(D)** Serial dilution (10-fold) of ctDNA (45% VAF) in wild-type cfDNA (median coverage 147,954; range 127,981–201438 reads). PCR was performed using the Q5 polymerase (NEB), 40 PCR cycles and 30 ng of cfDNA as PCR template. **(E)** NGS sensitivity for the detection of low-level ctDNA (0.1% VAF) in cfDNA samples (*n* = 25) using 5 or 20 ng of cfDNA as template for PCR amplification (median coverage 114,830; range 71,321–323,158 reads). Wild-type cfDNA (*n* = 9) was used to measure the specific false-positive rate (substitution error) for the detection of the *KRAS* c.35G > A variant. **(F)** Detection of ctDNA at the NGS cutoff for quantification (0.01% VAF) in cfDNA samples of CRC patients (*n* = 10) (median coverage 137,989; range 114,597–194,558 reads). Variant calling was performed using reads from a single PCR with 20 ng cfDNA input or by the integration of sequencing reads from pooled triplicate PCRs and 5 ng of cfDNA as template.

The collection of blood in conventional EDTA tubes resulted in a 10- to 100-fold increase in extracted DNA (after 3 and 14 days), indicating an enhanced release of genomic DNA from the cellular fraction (Figure 1A). Consequently, the detection of mutant alleles in EDTA collection tubes decreased significantly (*p* < 0.0001) during blood incubation (half-life of 12–30 h). Under these conditions, ctDNA was below detection limits (<0.01%) after 14 days of incubation. In contrast, concentrations of extracted DNA (mean 4.08–4.56 ng ml^−1^ plasma) and VAFs (mean 17.89–22.59%) of the *KRAS* c.38G > A variant (20% VAF) were stable for up to 96 h in Cell-Free DNA BCT (Streck) tubes, followed by a slight increase in DNA yield (mean 6.28 ng ml^−1^) and a concomitant decrease in *KRAS* VAFs (mean 14.64%) measured at day 14.

### 3.2 Comparison of Manual and Automated cfDNA Extraction Protocols

The evaluation of manual cfDNA extraction protocols revealed significant differences in yield and stability of ctDNA (Figure 1B). Total yields of cfDNA from plasma samples (1–3 ml) were significantly higher (*p* < 0.0001) using procedures by Zymo Research (median 7.01; 95% CI 5.69–8.03 ng ml^−1^) and Qiagen (median 6.47; 95% CI 4.98–8.05 ng ml^−1^) than those using the protocol of Analytik Jena (median 2.42, 95% CI 2.19–3.19 ng ml^−1^). Similarly, the detection of mutant alleles with the *KRAS* c.38G > A mutation at ∼10% VAF was most accurate using the kit by Zymo Research (median 8.78; 95% CI 7.33–10.30% VAF) for cfDNA preparation, followed by Qiagen (median 5.86; 95% CI 4.58–7.23% VAF) and AJ (median 3.96; 95% CI 3.09–5.62% VAF). No significant differences for cfDNA extraction yields (2.9–21.1 vs. 1.7–18.2 ng ml^−1^; *p* = 0.9807) and the detection of prevalent mutant alleles (0.09–3.84 vs. 0.06–4.35% VAF; *p* = 0.9629) were observed for the preparation of matched plasma samples from CRC patients (*n* = 15) using the manual protocol by Zymo Research and an automated procedure on a QIAsymphony instrument (Figure 1C).

### 3.3 NGS Sensitivity for the Detection of Low-Level ctDNA

The detection of the *KRAS* c.35G > A variant in cfDNA was linear (*r* ^2^ = 0.9964) between 0.0045 and 45% VAF (Figure 1D). The mean false-positive rate (substitution error) for the *KRAS* c.35G > A variant, measured in wild-type cfDNA, was 0.0016 ± 0.0007% (Figure 1E; Supplementary Table S2). Applying the empirical rule for normal distribution, 99.73% of data observed will range within three standard deviations of the mean. According to that estimate, the specific limit for detection of the *KRAS* c.35G > A transition mutation in plasma cfDNA would correspond to a VAF of 0.0037% and one mutant allele out of 27,027 wild-type alleles, respectively. This is clearly below the predefined cutoff (0.01% VAF) for SNV detection, previously determined for our error-reduced targeted NGS approach (Stasik et al., 2018). In line, *KRAS* mutant alleles (c.35G > A) at 0.1% VAF were detected with high sensitivity in 23/25 samples (94%) in cfDNA of CRC patient samples. Specificity for the *KRAS* c.35G > A variant at 0.1% VAF was 100% (Figure 1E). Quantification of mutant alleles non-significantly (*p* = 0.2000) increased using 20 ng of cfDNA for amplification (median 0.12; 95% CI 0.09–0.21% VAF) compared to an input of 5 ng cfDNA as a PCR template (median 0.08; 95% CI 0.06–0.15% VAF) (Figure 1E). The detection of low-level ctDNA in 10 samples with the *KRAS* c.35G > A variant at ∼0.01% VAF increased 1.5-fold (60–90%) by the integration of sequencing reads from pooled triplicate PCRs as compared to the analysis of a single PCR with higher amounts of cfDNA added per sample (Figure 1F).

### 3.4 Retrospective Validation of ctDNA-Based Detection of Recurrence

**FIGURE 2:**
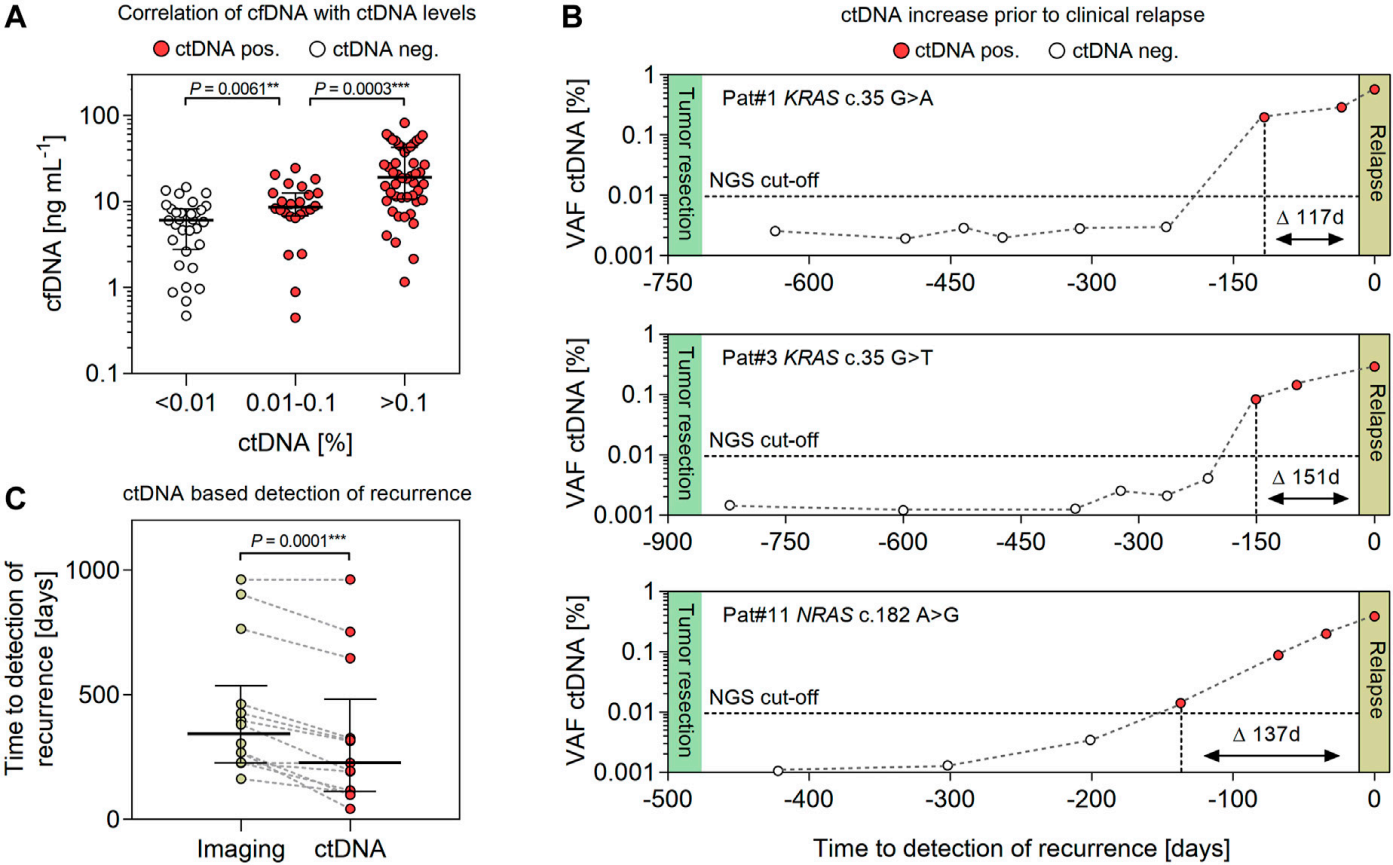
Evaluating the feasibility of detecting ctDNA for early prediction of relapse in CRC patients. **(A)** Association of cfDNA with levels of ctDNA (<0.01%, 0.01–0.1% and >0.1%) in plasma samples (*n* = 104) of CRC patients (*n* = 14). **(B)** Exemplary illustration of ctDNA dynamics in *RAS*-mutated CRC patients during adjuvant therapy post-tumor resection. The NGS detection limit is indicated at 0.01% VAF. **(C)** Comparing the progression free survival (PFS) of CRC patients (*n* = 14) assessed by conventional imaging-based diagnostics and NGS-based detection of ctDNA. Error bars represent median values and interquartile range.

A total of 14 patients (10 males and 4 females) with advanced colorectal cancer (stages II–IV) and relapse during adjuvant therapy post-tumor resection were enrolled. The median age at tumor resection was 68 (range 50–81) years. Resected primary tumor samples were found positive for oncogenic hot spot mutations in *KRAS* (*n* = 9), *NRAS* (*n* = 2) and *TP53* (*n* = 3). The median follow-up until imaging-based detection of recurrence was 381 (range 163–962) days. Demographic characteristics and pathological findings of patients are summarized in Table 1. In matched plasma cfDNA, tumor-specific point mutations were detected in 13/14 patients (92.85%) at the time of imaging-based detection of relapse (Table 1). No ctDNA was detected in one patient (Pat#5) with a *KRAS* c.34G > T variant identified with 9% VAF in the primary tumor. In general, levels of ctDNA increased with concentrations of total cfDNA, pointing to a correlation of cfDNA levels with tumor burden (Figure 2A). Significant differences were measured for levels of plasma cfDNA in patients without detectable ctDNA <0.01%, low-level ctDNA 0.01–0.1% (*p* = 0.0061), and ctDNA >0.1% (*p* = 0.0003) (median cfDNA 6.07, 8.59, and 19.18 ng ml^−1^ plasma). For all patients monitored, ctDNA was below detection limits (<0.01%) in samples <1 month post-tumor resection, demonstrating initial clearance of tumor-derived mutant alleles in blood circulation. Prior to clinical relapse, accumulation of ctDNA increased with daily rates of 0.003–0.012% (ctDNA d-1) (Figure 2B). The highest concentrations of plasma ctDNA were measured at the time of imaging-based detection of relapse, with a median VAF of 0.38% (range 0.18–5.04%). The median delta between imaging-based and ctDNA-based detection of recurrence was 112 days (*p* < 0.0001) (range 0–226 days) (Table 1; Figure 2C).

**TABLE 1:** Retrospective validation of ctDNA-based detection of recurrence.

Patient characteristic			TNM classification					ctDNA marker		Time to detection of recurrence [d]		
Pat	Gender	Age (years)	UICC	pT	pN	M	Gene	HGVS	p.HGVS	Imaging	ctDNA	∆
Pat#1	Male	77	IV	4	2	1	*KRAS*	c.35G > A	p.Gly12Asp	764	647	117
Pat#2	Male	66	IV	4	2	1	*KRAS*	c.35G > A	p.Gly12Asp	962	962	0
Pat#3	Female	81	III	3	1	0	*KRAS*	c.35G > T	p.Gly12Val	903	752	151
Pat#4	Female	53	IV	3	0	1	*KRAS*	c.35G > T	p.Gly12Val	226	226	0
Pat#5	Female	62	II	4	0	0	*KRAS*	c.34G > T	p.Gly12Cys	305	n.d	n.a
Pat#6	Female	72	IV	3	1	1	*KRAS*	c.40G > A	p.Val14Ile	163	109	54
Pat#7	Male	74	III	3	1	0	*KRAS*	c.175G > A	p.Ala59Thr	268	42	226
Pat#8	Male	63	III	3	1	0	*KRAS*	c.436G > A	p.Ala146Thr	228	116	112
Pat#9	Male	70	III	3	2	0	*KRAS*	c.350A > G	p.Lys117Arg	397	315	82
Pat#10	Male	54	IV	3	2	1	*NRAS*	c.38G > A	p.Gly13Asp	227	192	35
Pat#11	Male	50	IV	4	1	1	*NRAS*	c.182A > G	p.Gln61Arg	463	326	137
Pat#12	Male	54	IV	4	2	1	*TP53*	c.584T > C	p.Ile195Thr	427	318	109
Pat#13	Male	77	IV	4	1	1	*TP53*	c.743G > A	p.Arg248Gln	269	98	171
Pat#14	Male	70	IV	3	1	1	*TP53*	c.742C > T	p.Arg248Trp	381	195	186

Abbreviations: UICC (Union for International Cancer Control); TNM (tumor (T), node (N), and metastasis (M)); HGVS (Human Genome Variation Society); ∆ (difference between the detection of tumor progression between methods applied).

### 3.5 Prospective Evaluation of ctDNA Persistence in Postoperative Samples

**FIGURE 3:**
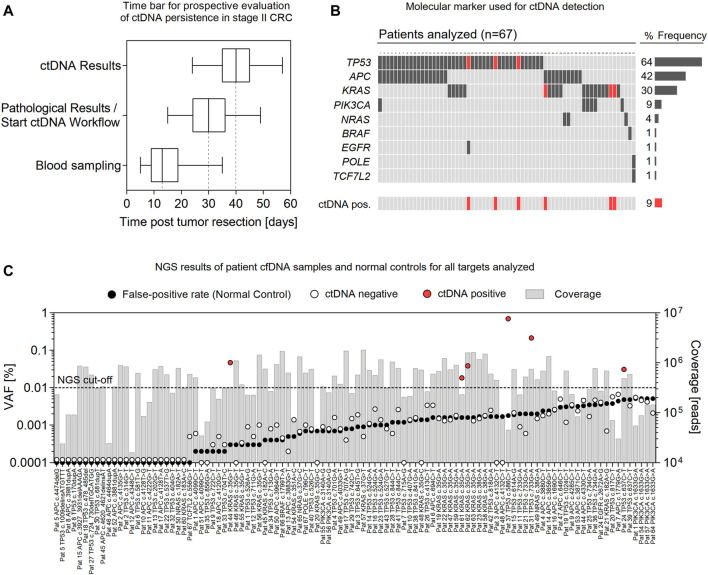
Rapid prospective evaluation of ctDNA persistence in stage II CRC. **(A)** Time bar showing intervals of blood sampling, pathological analysis of primary tumor material, and ctDNA analysis post-tumor resection of stage II CRC patients (*n* = 67). Box plots represent median values with interquartile range; Box–Whiskers represent min. to max. values. **(B)** Molecular marker (and frequency) used for ctDNA analysis and samples positive for ctDNA (red). **(C)** NGS results of all ctDNA targets analyzed (*n* = 104) and corresponding false-positive rates (black) of individual mutations. Bars represent the sequencing coverage of each sample. ctDNA positive samples are presented in red.

For the prospective evaluation of postoperative ctDNA detection, 67 patients (CRC stage II, MSI low, median age of 69 years; range 27–85 years) were included. Postoperative blood sampling was conducted at a median of 13 (range 5–35) days after surgery (Figure 3A). Pathological findings of primary tumor material and results of the ctDNA analysis were available at a median of 30 (range 15–49) days and 40 (range 24–57) days after tumor resection, respectively. The most common molecular alterations used for ctDNA analysis were mutations in *TP53* (64%), *APC* (42%), and *KRAS* (30%) (Figure 3B). For roughly half of patients (48%), one single molecular marker was used for ctDNA screening, while two (or three in one case) eligible mutations were targeted in 52% of patients. Out of the 67 patients, deep sequencing of cfDNA samples (median coverage of 486,240, range 15,986–1,806,561 reads per target) revealed the presence of postoperative ctDNA in 6 patients (9%), with a median VAF of 0.042% (range 0.018–0.697%) (Figure 3C; Supplementary Table S2). Generally, false-positive rates were below 0.01% VAF for all mutations analyzed (Figure 3C; Supplementary Table S2). However, substantial differences were measured for false-positive rates of individual mutations, which in part limits or qualifies their suitability as sensitive ctDNA markers. For example, background error was significantly lower for transversion mutations (median 0.0003, range 0–0.0024%) and insertion/deletion variants (median 0) than for transition mutations (median 0.0016; range 0–0.0051%) with the highest false-positive rate measured for the PIK3CA c.1633G > A variant (mean 0.0051 ± 0.0012% StDiv) (Supplementary Figure S1; Supplementary Table S2).

## 4 Discussion

In this study, we provide an easily adaptable, cost-effective, and rapid approach for the sensitive detection of plasma ctDNA in clinical routine, using adequate pre-analytical cfDNA processing and subsequent error-reduced deep sequencing. In addition, we document the translation of this approach for rapid prospective evaluation of ctDNA persistence in stage II CRC and the implementation of ctDNA for early prediction of disease recurrence.

The potential of ctDNA analysis as liquid biopsy in CRC for somatic tumor profiling (Strickler et al., 2018), the detection of treatment response (Siravegna et al., 2015; Tie et al., 2015; Reinert et al., 2016; Schøler et al., 2017; Tie et al., 2019), and disease progression (El Messaoudi et al., 2016; Tie et al., 2016; Reinert et al., 2019; Wang et al., 2019; Luo et al., 2020; Yeh et al., 2020) has been outlined previously. Typically, 15–20% of stage II CRC patients with curative resection will face disease recurrence (Böckelman et al., 2015), highlighting the need for sensitive monitoring strategies. We demonstrate an overall ctDNA persistence of ∼9% in stage II CRC patients after surgery, which confirms recent data on the frequency of postoperative ctDNA persistence (Tie et al., 2016; Reinert et al., 2019). In addition, tumor-informed ctDNA was detected 1–8 months prior to imaging-based detection of progression in 92.85% of CRC patients with relapse, which is in the range or superior to rates (72–92.3%) determined previously (Tie et al., 2015; Schøler et al., 2017; Reinert et al., 2019; Wang et al., 2019; Luo et al., 2020; Yeh et al., 2020). Interestingly, in two patients (Pat. 2 and 4), no difference was observed between ctDNA- and imaging-based detection of progression. However, for these patients, no plasma samples were available within a period of 5 months prior to clinical relapse in our retrospective analysis, emphasizing the importance of appropriate sampling intervals (i.e., every 2 months) for monitoring. The only patient (Pat. 5) without detectable ctDNA at relapse carried a *KRAS* hot spot mutation found at subclonal levels in the primary tumor tissue, pointing to the presence of intra-tumoral heterogeneity and clonal selection during cancer progression (Khan et al., 2018). Likewise, a negative selection of *RAS* mutations due to selective pressure after treatment has been observed in hematological malignancies (Oshima et al., 2016) and in the plasma of CRC patients (Spindler et al., 2014), with implications for *RAS* testing during adjuvant therapy (Thierry et al., 2017; Normanno et al., 2018). In this regard, the application of whole gene panels for ctDNA monitoring can improve the detection of emerging CRC clones and the assessment of clonal tumor evolution, which might minimize false-negative ctDNA results but increases costs and analysis time to achieve sufficient sensitivity (Malapelle et al., 2017). Moreover, false-negative postoperative ctDNA results might occur in patients with low-shedding tumors, which can be excluded from ctDNA-guided treatment by analyzing presurgery blood samples (Naidoo et al., 2021). In addition to biological tumor features, the sensitivity of most ctDNA assays is limited by the available cfDNA template, which in turn depends on efficient extraction procedures (Barták et al., 2019; van der Leest et al., 2020).

In our study, adequate cfDNA processing routinely allowed the extraction of ∼10 ng of cfDNA mL^−1^ plasma in clinical samples of CRC patients (depending on tumor load), which is in the range of concentrations typically measured for cfDNA in human plasma (Fleischhacker and Schmidt, 2007). As we used 2.5 ml plasma for cfDNA extraction, 8,333 alleles per patient sample were available for PCR amplification (assuming ∼3 pg of DNA per haploid human genome and 10 ng cfDNA mL^−1^ plasma), which results in a theoretical limit of 0.012% VAF for the quantification of heterozygous variants. In line, sensitivity for low-level ctDNA increased by increasing the input of cfDNA as PCR template and by the integration of sequencing reads from triplicate PCRs, pointing to an “all-or-nothing” positive amplification in samples with low mutant DNA copy numbers and a potential benefit of binning for barcoded sequencing reads. In combination with sufficient sequencing depth (≥100.000 reads), the sensitivity of our error-reduced NGS approach (0.01% VAF) is one to two orders of magnitude higher than conventional targeted NGS procedures (typically 0.1–1% error) (Rachiglio et al., 2016; Osumi et al., 2019) and similar to sensitivities determined for digital droplet PCR (Demuth et al., 2018) or for other modified NGS protocols (Yeh et al., 2020). Turnaround time for analysis (24–48 h) and costs (∼45€ per sample) of our NGS-based procedure are slightly higher than ddPCR (8 h, <20€) and are in the range of other PCR-based approaches with similar sensitivities for cfDNA analysis such as BEAMing (Demuth et al., 2018; Garcia et al., 2018). However, in comparison to other ctDNA assays (Malapelle et al., 2017; Kastrisiou et al., 2019), our targeted NGS procedure allows a versatile (i.e., simple multiplexing) analysis of ctDNA, which is relevant for applied clinical diagnostics.

From the theoretical considerations outlined earlier, a further increase in sensitivity to 0.001% VAF would require template concentrations of ∼300 ng of cfDNA (100.000 haploid copies) for PCR-based methods tracking a single molecular marker, which is likely unrealistic using common volumes of whole blood (10 ml) for cfDNA preparation. Recently, a patient-specific sequencing approach integrating variant reads of multiple mutated genomic loci reported on sensitivities of 0.001–0.0001% for ctDNA monitoring, which in part depended on tumor mutational burden and high plasma input material (Wan et al., 2020). Accordingly, the parallel analysis of multiple ctDNA markers (i.e., up to 3 markers per patient in our prospective study) offers additional capacity to increase sensitivity for clinical implementation. Based on the methodological improvements and interim results in the present study, a multicenter, prospective, randomized trial (CIRCULATE, NCT04089631, phase III), which started in June 2020, is currently evaluating circulating tumor DNA-based decision for randomized adjuvant treatment in colon cancer stage II (Folprecht et al., 2020).

Taken together, we provide a robust and easily adaptable approach for the sensitive detection of plasma ctDNA in clinical routine, using optimized pre-analytical workup of samples for efficient cfDNA preparation and subsequent error-reduced targeted deep sequencing. Using optimized conditions, the detection of postoperative ctDNA for early prediction of CRC persistence or recurrence is feasible with excellent sensitivity and specificity at frequencies in the range of 0.01–0.1% and superior compared to standard imaging-based surveillance.

## Data Availability

The original contributions presented in the study are included in the article/supplementary materials, further inquiries can be directed to the corresponding author.
