# Supplementary material for: Sensitive Quantification of Cell-Free Tumor DNA for Early Detection of Recurrence in Colorectal Cancer
Source: Front Genet. 2022 Jan 5;12:811291. doi: 10.3389/fgene.2021.811291 (PMC8766716; doi:10.3389/fgene.2021.811291)
Supplement: Supplementary file 1 [file DataSheet1.DOCX]

Supplementary Material

# Supplementary Figures and Tables

| **Table S1 Primer sequences, annealing temperatures, fragment length and genomic positions** | | | | |
| --- | --- | --- | --- | --- |
| Target* | Primer (target specific sequence) 5´-3´ | Annealing | Amplicon length** | Genomic Position |
| *APC* (E5) | (F) TTGAACTGACCCCAATTTGTT | 63°C | 304 | chr5:112127972-112128210 |
|  | (R) TGGGACTGTAAAAGCTGTCG |  |  |  |
| *APC* (E12) | (F) AATTTGCAGGTTATTGCGAGT | 65°C | 290 | chr5:112164544-112164768 |
|  | (R) CACAGGTAAGAAATTAGGAAATCTCA |  |  |  |
| *APC* (E13) | (F) TTTCTTACTAGGAATCAACCCTCAA | 64°C | 291 | chr5:112170637-112170863 |
|  | (R) CCTGTGGTCCTCATTTGTAGC |  |  |  |
| *APC* (E14.1) | (F) TCAGCTGAAGATGAAATAGGATG | 63°C | 233 | chr5:112175135-112175303 |
|  | (R) GCAGTCTGCTGGATTTGGTT |  |  |  |
| *APC* (E14.2) | (F) GATCCTGTGAGCGAAGTTCC | 63°C | 264 | chr5:112175243-112175442 |
|  | (R) AACATGAGTGGGGTCTCCTG |  |  |  |
| *APC* (E14.3) | (F) CCAAAAGTGGTGCTCAGACA | 63°C | 273 | chr5:112175376-112175584 |
|  | (R) CATGGTTTGTCCAGGGCTAT |  |  |  |
| *APC* (E14.4) | (F) GGCATTATAAGCCCCAGTGA | 66°C | 274 | chr5:112175537-112175746 |
|  | (R) AGCATCTGGAAGAACCTGGA |  |  |  |
| *APC* (E14.5) | (F) GGACCTAAGCAAGCTGCAGTA | 62°C | 272 | chr5:112175687-112175894 |
|  | (R) TCCCATTGTCATTTTCCTGA |  |  |  |
| *APC* (E14.6) | (F) TGGAATTAAGAATAATGCCTCCA | 62°C | 261 | chr5:112175850-112176046 |
|  | (R) TGTTGGCATGGCAGAAATAA |  |  |  |
| *BRAF* (E15) | (F) CCTTTACTTACTACACCTCAGA | 61°C | 193 | chr7:140453088-140453214 |
|  | (R) GATCCAGACAACTGTTCAAACT |  |  |  |
| *KRAS* (E2) | (F) ATAAACTTGTGGTAGTTGGAGC | 63°C | 168 | chr12:25398207-25398308 |
|  | (R) CCTCTATTGTTGGATCATATTCGTC |  |  |  |
| *KRAS* (E3) | (F) ATAAACTTGTGGTAGTTGGAGC | 63°C | 168 | chr12:25380236-25380336 |
|  | (R) CCTCTATTGTTGGATCATATTCGTC |  |  |  |
| *KRAS* (E4.1) | (F) ACAGGCTCAGGACTTAGCAAGAAGT | 63°C | 226 | chr12:25378457-25378614 |
|  | (R) AGCATAATTGAGAGAAAAACTGA |  |  |  |
| *KRAS* (E4.2) | (F) CCTGTCTTGTCTTTGCTGATG | 63°C | 294 | chr12:25378547-25378775 |
|  | (R) TTGTGGACAGGTTTTGAAAGA |  |  |  |
| *NRAS* (E2) | (F) CAGGTTCTTGCTGGTGTGAA | 66°C | 172 | chr1:115258702-115258801 |
|  | (R) TGGTTCTGGATTAGCTGGATTG |  |  |  |
| *NRAS* (E3) | (F) TAAAAATTGAACTTCCCTCCCTCCCTGC | 66°C | 328 | chr1:115256391-115256650 |
|  | (R) TCCTTTCAGAGAAAATAATGCTCCTAG |  |  |  |
| *PIK3CA* (E10) | (F) TCCAGAGGGGAAAAATATGAC | 62°C | 275 | chr3:178935941-178936150 |
|  | (R) AACAGAGAATCTCCATTTTAGCA |  |  |  |
| *PIK3CA* (E21) | (F) TTCTCAATGATGCTTGGCTCT | 64°C | 302 | chr3:178951949-178952185 |
|  | (R) CCAGAGTGAGCTTTCATTTTCTC |  |  |  |
| *POLE* (E8) | (F) GGATGAAGGTAACACAAGCAAA | 64°C | 244 | chr12:133253921-133254099 |
|  | (R) TGGGTGAATCCACAGAAGAA |  |  |  |
| *TCF7L2* (E5) | (F) CTCTGTTCTCCTCCCCACAG | 66°C | 282 | chr10:114900923-114901139 |
|  | (R) CTCAAAGGTTCCCCCAGAAC |  |  |  |
| *TP53* (E4) | (F) AACTGACCGTGCAAGTCACA | 67°C | 312 | chr17:7579305-7579550 |
|  | (R) CCCCGGACGATATTGAACAATG |  |  |  |
| *TP53* (E5) | (F) TTCAACTCTGTCTCCTTCCTCTTCCTAC | 70°C | 316 | chr17:7578334-7578584 |
|  | (R) AACCAGCCCTGTCGTCTCTCCA |  |  |  |
| *TP53* (E6) | (F) ACCACCCTTAACCCCTCCT | 67°C | 256 | chr17:7578131-7578321 |
|  | (R) CAGGCCTCTGATTCCTCACT |  |  |  |
| *TP53* (E7) | (F) GTGTGCAGGGTGGCAAGT | 66°C | 226 | chr17:7577471-7577631 |
|  | (R) CTTGGGCCTGTGTTATCTCC |  |  |  |
| *TP53* (E8) | (F) TTCCTTACTGCCTCTTGCTTCTCTT | 69°C | 277 | chr17:7576985-7577196 |
|  | (R) GTCTCCTCCACCGCTTCTTGTC |  |  |  |
| *TP53* (E10) | (F) GGAGTAGGGCCAGGAAGG | 65°C | 314 | chr17:7573893-7574141 |
|  | (R) TGCATGTTGCTTTTGTACCG |  |  |  |
| * Targets were amplified using 5 ng template cfDNA and Q5 polymerase with amplification thermocycle consisting of: | | | | |
| 98°C for 30 sec, followed by 40 cycles at 98°C for 5 s, XX°C for 10 sec, and 72°C for 20 sec, final extension at 72°C for | | | | |
| 2 min |  |  |  |  |
| ** Fragment length is given including barcodes, Ion A and truncated P1 (trP1) adapter sequences | | | |  |

| **Table S2 NGS-based prospective evaluation of ctDNA persistence in stage II CRC patients** | | | | | | | | | |
| --- | --- | --- | --- | --- | --- | --- | --- | --- | --- |
| Pat. | ctDNA Marker | VAF Primary Tumor [%] | Mutation Type | ctDNA VAF [%] | Coverage [reads] | False-positive Rate [%] | StDiv [%] | NGS Cut-Off* [%] | ctDNA Status** |
| 1 | *APC* c.4099C>T | 29.77 | transition | 0.0006 | 503316 | 0.0002 | 0.0002 | 0.0007 | negative |
| 1 | *PIK3CA* c.1633G>A | 20.20 | transition | 0.0055 | 54327 | 0.0051 | 0.0012 | 0.0087 | negative |
| 1 | *TP53* c.526A>G | 56.07 | transition | 0.0009 | 223176 | 0.0003 | 0.0003 | 0.0012 | negative |
| 2 | *APC* c.4135G>T | 33.44 | transversion | 0.0000 | 883269 | 0.0000 | 0.0000 | 0.0000 | negative |
| 2 | *TP53* c.314G>T | 57.64 | transversion | 0.0000 | 832877 | 0.0000 | 0.0000 | 0.0000 | negative |
| 3 | *APC* c.4132C>T | 38.48 | transition | 0.0000 | 445735 | 0.0017 | 0.0004 | 0.0031 | negative |
| 3 | *TP53* c.645T>G | 48.35 | transversion | 0.0019 | 566903 | 0.0009 | 0.0009 | 0.0036 | negative |
| 4 | *APC* c.3880C>T | 32.84 | transition | 0.0018 | 681555 | 0.0024 | 0.0009 | 0.0051 | negative |
| 4 | *TP53* c.701G>A | 34.13 | transition | 0.0016 | 541063 | 0.0007 | 0.0007 | 0.0028 | negative |
| 5 | *APC* c.4474delG | 46.31 | InDel | 0.0000 | 230065 | 0.0000 | 0.0000 | 0.0000 | negative |
| 5 | *TP53* c.1009delinsATGTTT | 83.02 | InDel | 0.0000 | 23698 | 0.0000 | 0.0000 | 0.0000 | negative |
| 6 | *APC* c.694C>T | 18.50 | transition | 0.0034 | 593395 | 0.0016 | 0.0011 | 0.0049 | negative |
| 6 | *TP53* c.581T>G | 38.78 | transversion | 0.0000 | 613704 | 0.0000 | 0.0000 | 0.0000 | negative |
| 7 | *APC* c.1779G>A | 25.36 | transition | 0.0067 | 29933 | 0.0044 | 0.0012 | 0.0080 | negative |
| 7 | *TP53* c.715A>G | 35.12 | transition | 0.0000 | 204087 | 0.0012 | 0.0009 | 0.0039 | negative |
| 8 | *APC* c.3981dupA | 73.6 | InDel | 0.0000 | 90000 | 0.0000 | 0.0000 | 0.0000 | negative |
| 8 | *TP53* c.170dupA | 63.9 | InDel | 0.0000 | 90000 | 0.0000 | 0.0000 | 0.0000 | negative |
| 9 | *APC* c.4285C>T | 36.33 | transition | 0.0035 | 231719 | 0.0032 | 0.0014 | 0.0074 | negative |
| 9 | *TP53* c.722C>T | 58.11 | transition | 0.0003 | 308560 | 0.0002 | 0.0002 | 0.0008 | negative |
| 10 | *APC* c.4128T>G | 58.21 | transversion | 0.0000 | 84168 | 0.0000 | 0.0000 | 0.0000 | negative |
| 10 | *TP53* c.407G>A | 46.98 | transition | 0.0014 | 211434 | 0.0013 | 0.0004 | 0.0025 | negative |
| 11 | *APC* c.4222G>T | 22.67 | transversion | 0.0000 | 162842 | 0.0000 | 0.0000 | 0.0000 | negative |
| 11 | *TP53* c.733G>A | 43.48 | transition | 0.0009 | 229677 | 0.0020 | 0.0020 | 0.0078 | negative |
| 12 | *APC* c.4348C>T | 29.98 | transition | 0.0000 | 15986 | 0.0000 | 0.0000 | 0.0000 | negative |
| 12 | *TP53* c.711G>A | 24.75 | transition | 0.0005 | 223944 | 0.0003 | 0.0003 | 0.0012 | negative |
| 13 | *APC* c.3883G>T | 73.1 | transversion | 0.0002 | 621021 | 0.0005 | 0.0002 | 0.0011 | negative |
| 13 | *TP53* c.202G>T | 60.2 | transversion | 0.0000 | 729170 | 0.0000 | 0.0000 | 0.0000 | negative |
| 14 | *APC* c.3856G>T | 66.9 | transversion | 0.0020 | 1335816 | 0.0024 | 0.0011 | 0.0057 | negative |
| 14 | *TP53* c.524G>A | 55.9 | transition | 0.0011 | 704620 | 0.0010 | 0.0007 | 0.0031 | negative |
| 15 | *APC* c.3927delAAAGA | 27.6 | InDel | 0.0000 | 865960 | 0.0000 | 0.0000 | 0.0000 | negative |
| 15 | *TP53* c.614A>G | 32.12 | transition | 0.0019 | 736173 | 0.0019 | 0.0008 | 0.0043 | negative |
| 16 | *APC* c.1690C>T | 28.5 | transition | 0.0026 | 273768 | 0.0025 | 0.0009 | 0.0052 | negative |
| 16 | *TP53* c.524G>A | 71.2 | transition | 0.0027 | 561400 | 0.0010 | 0.0007 | 0.0031 | negative |
| 17 | *APC* c.4128T>A | 75.2 | transversion | 0.0000 | 837384 | 0.0000 | 0.0000 | 0.0000 | negative |
| 17 | *TP53* c.707A>G | 60.2 | transition | 0.0004 | 1721212 | 0.0008 | 0.0006 | 0.0026 | negative |
| 18 | *APC* c.4120G>T | 18.5 | transversion | 0.0005 | 186881 | 0.0002 | 0.0002 | 0.0008 | negative |
| 18 | *TP53* c.478del | 64.6 | InDel | 0.0000 | 818449 | 0.0000 | 0.0000 | 0.0000 | negative |
| 19 | *KRAS* c.35G>A | 48.95 | transition | 0.0015 | 669073 | 0.0016 | 0.0007 | 0.0036 | negative |
| 19 | *TP53* c.1024C>T | 51.31 | transition | 0.0012 | 683912 | 0.0031 | 0.0009 | 0.0058 | negative |
| 20 | *KRAS* c.35G>C | 29.79 | transversion | 0.0000 | 44281 | 0.0007 | 0.0007 | 0.0028 | negative |
| 20 | *TP53* c.817C>T | 43.8 | transition | 0.0057 | 70321 | 0.0038 | 0.0015 | 0.0083 | negative |
| 21 | *KRAS* c.182A>G | 66.44 | transition | 0.0007 | 686906 | 0.0038 | 0.0010 | 0.0068 | negative |
| 21 | *TP53* c.733G>A | 46.49 | transition | 0.0006 | 519015 | 0.0020 | 0.0020 | 0.0078 | negative |
| 22 | *KRAS* c.35G>A | 31.59 | transition | 0.0013 | 1230328 | 0.0016 | 0.0007 | 0.0036 | negative |
| 22 | *TP53* c.537T>G | 44.72 | transversion | 0.0000 | 576198 | 0.0000 | 0.0000 | 0.0000 | negative |
| 23 | *KRAS* c.38G>A | 24.6 | transition | 0.0015 | 1164365 | 0.0017 | 0.0002 | 0.0023 | negative |
| 23 | *TP53* c.524G>A | 34.8 | transition | 0.0014 | 1144123 | 0.0010 | 0.0007 | 0.0031 | negative |
| 24 | *EGFR* c.2672A>G | 25.64 | transition | 0.0041 | 268366 | 0.0036 | 0.0009 | 0.0063 | negative |
| **24** | ***TP53* c.637C>T** | **28.89** | **transition** | **0.0308** | **486240** | **0.0048** | **0.0008** | **0.0072** | **positive** |
| 25 | *TP53* c.734G>A | 65.46 | transition | 0.0017 | 289192 | 0.0035 | 0.0006 | 0.0054 | negative |
| 26 | *TP53* c.413C>T | 6.05 | transition | 0.0034 | 263021 | 0.0014 | 0.0010 | 0.0044 | negative |
| 27 | *TP53* c.724delTGCATGG | 14.48 | InDel | 0.0000 | 572108 | 0.0000 | 0.0000 | 0.0000 | negative |
| 28 | *TP53* c.844C>T | 48.31 | transition | 0.0006 | 1029483 | 0.0012 | 0.0007 | 0.0032 | negative |
| 29 | *TP53* c.742C>T | 60.50 | transition | 0.0015 | 243823 | 0.0008 | 0.0005 | 0.0023 | negative |
| 30 | *TP53* c.681dupT | 52.48 | InDel | 0.0000 | 310137 | 0.0000 | 0.0000 | 0.0000 | negative |
| **31** | ***TP53* c.733G>A** | **45.29** | **transition** | **0.2137** | **738742** | **0.0020** | **0.0020** | **0.0078** | **positive** |
| 32 | *TP53* c.404G>T | 47.96 | transversion | 0.0000 | 245119 | 0.0000 | 0.0000 | 0.0000 | negative |
| 33 | *TP53* c.704T>C | 63.26 | transversion | 0.0003 | 313309 | 0.0002 | 0.0002 | 0.0008 | negative |
| 34 | *TP53* c.713G>A | 52.21 | transition | 0.0008 | 264740 | 0.0004 | 0.0003 | 0.0013 | negative |
| 35 | *TP53* c.856G>A | 51.4 | transition | 0.0000 | 65024 | 0.0002 | 0.0001 | 0.0005 | negative |
| 36 | *TP53* c.734G>A | 31.51 | transition | 0.0047 | 766239 | 0.0035 | 0.0006 | 0.0054 | negative |
| **37** | ***TP53* c.586C>T** | **56.95** | **transition** | **0.6978** | **44855** | **0.0018** | **0.0004** | **0.0030** | **positive** |
| 38 | *TP53* c.841G>A | 47.3 | transition | 0.0014 | 770501 | 0.0014 | 0.0009 | 0.0041 | negative |
| 39 | *TP53* c.637C>T | 61 | transition | 0.0033 | 582093 | 0.0048 | 0.0008 | 0.0072 | negative |
| 40 | *TP53* c.526C>T | 50.3 | transition | 0.0007 | 743320 | 0.0007 | 0.0001 | 0.0010 | negative |
| 41 | *TP53* c.844C>T | 24.77 | transition | 0.0026 | 778859 | 0.0012 | 0.0007 | 0.0032 | negative |
| 42 | *TP53* c.818G>A | 72.9 | transition | 0.0025 | 596857 | 0.0017 | 0.0007 | 0.0038 | negative |
| 43 | *TP53* c.527G>T | 54.15 | transversion | 0.0008 | 309091 | 0.0010 | 0.0002 | 0.0016 | negative |
| 44 | *APC* c.4330C>T | 6.93 | transition | 0.0046 | 195911 | 0.0033 | 0.0011 | 0.0066 | negative |
| **44** | ***KRAS* c.35G>T** | **42.46** | **transversion** | **0.0468** | **318607** | **0.0003** | **0.0002** | **0.0009** | **positive** |
| 45 | *APC* c.4620delinsAT | 32.12 | InDel | 0.0000 | 333959 | 0.0000 | 0.0000 | 0.0000 | negative |
| 45 | *KRAS* c.182A>C | 30.81 | transversion | 0.0000 | 745953 | 0.0004 | 0.0001 | 0.0007 | negative |
| 46 | *APC* c.4464dupA | 37.38 | InDel | 0.0000 | 24375 | 0.0000 | 0.0000 | 0.0000 | negative |
| 46 | *KRAS* c.35G>T | 33.77 | transversion | 0.0000 | 1099635 | 0.0003 | 0.0002 | 0.0009 | negative |
| 47 | *APC* c.646C>T | 41.6 | transition | 0.0050 | 141848 | 0.0030 | 0.0009 | 0.0057 | negative |
| 47 | *KRAS* c.35G>A | 31.43 | transition | 0.0014 | 726651 | 0.0016 | 0.0007 | 0.0036 | negative |
| 48 | *APC* c.4132C>T | 12.49 | transition | 0.0000 | 75963 | 0.0017 | 0.0004 | 0.0031 | negative |
| 48 | *KRAS* c.35G>C | 18.56 | transversion | 0.0000 | 716743 | 0.0014 | 0.0003 | 0.0023 | negative |
| 49 | *APC* c.4033G>T | 18.43 | transversion | 0.0008 | 373397 | 0.0007 | 0.0001 | 0.0010 | negative |
| 49 | *NRAS* c.38G>A | 9.72 | transition | 0.0015 | 455634 | 0.0020 | 0.0016 | 0.0068 | negative |
| 50 | *APC* c.3964G>T | 19.09 | transversion | 0.0010 | 920346 | 0.0004 | 0.0003 | 0.0013 | negative |
| 50 | *NRAS* c.182A>T | 18.94 | transversion | 0.0000 | 780243 | 0.0000 | 0.0000 | 0.0000 | negative |
| 51 | *APC* c.4099C>T | 26.51 | transition | 0.0000 | 34207 | 0.0002 | 0.0002 | 0.0007 | negative |
| 52 | *APC* c.3881dupA | 42.90 | InDel | 0.0000 | 330470 | 0.0000 | 0.0000 | 0.0000 | negative |
| 53 | *APC* c.3871C>T | 20.12 | transition | 0.0024 | 83338 | 0.0032 | 0.0014 | 0.0074 | negative |
| 54 | *KRAS* c.351A>C | 27.45 | transversion | 0.0014 | 72410 | 0.0005 | 0.0003 | 0.0014 | negative |
| 54 | *PIK3CA* c.1633G>A | 11.78 | transition | 0.0046 | 87082 | 0.0051 | 0.0012 | 0.0087 | negative |
| 55 | *KRAS* c.35G>T | 18.64 | transversion | 0.0003 | 362074 | 0.0003 | 0.0002 | 0.0009 | negative |
| 55 | *PIK3CA* c.3140A>G | 20.95 | transition | 0.0009 | 331858 | 0.0007 | 0.0003 | 0.0016 | negative |
| 56 | *KRAS* c.35G>T | 43.6 | transversion | 0.0010 | 1423401 | 0.0003 | 0.0003 | 0.0012 | negative |
| 56 | *PIK3CA* c.3140A>G | 34.2 | transition | 0.0008 | 252304 | 0.0007 | 0.0003 | 0.0016 | negative |
| 57 | *KRAS* c.37G>T | 61.5 | transversion | 0.0005 | 1806561 | 0.0009 | 0.0000 | 0.0009 | negative |
| 57 | *PIK3CA* c.1633G>A | 47.2 | transition | 0.0042 | 142735 | 0.0051 | 0.0012 | 0.0087 | negative |
| 58 | *KRAS* c.38G>A | 63.58 | transition | 0.0018 | 1499274 | 0.0017 | 0.0002 | 0.0023 | negative |
| 59 | *KRAS* c.35G>A | 23.09 | transition | 0.0018 | 886971 | 0.0016 | 0.0007 | 0.0036 | negative |
| 60 | *KRAS* c.183A>C | 26.85 | transversion | 0.0000 | 304681 | 0.0000 | 0.0000 | 0.0000 | negative |
| **61** | ***KRAS* c.35G>A** | **33.88** | **transition** | **0.0183** | **229138** | **0.0016** | **0.0007** | **0.0036** | **positive** |
| **62** | ***KRAS* c.35G>A** | **25.45** | **transition** | **0.0384** | **1592064** | **0.0016** | **0.0007** | **0.0036** | **positive** |
| 63 | *KRAS* c.35G>A | 23.7 | transition | 0.0013 | 1599453 | 0.0016 | 0.0007 | 0.0036 | negative |
| 64 | *PIK3CA* c.1633G>A | 38.52 | transition | 0.0021 | 143710 | 0.0051 | 0.0012 | 0.0087 | negative |
| 65 | *NRAS* c.37G>C | 25.59 | transversion | 0.0006 | 1324871 | 0.0006 | 0.0005 | 0.0021 | negative |
| 66 | *BRAF* c.1799T>A | 32.58 | transversion | 0.0004 | 1688674 | 0.0004 | 0.0001 | 0.0007 | negative |
| 67 | *POLE* c.796C>T | 29.23 | transition | 0.0010 | 623676 | 0.0007 | 0.0005 | 0.0022 | negative |
| 67 | *TCF7L2* c.556G>T | 28.29 | transversion | 0.0005 | 587082 | 0.0001 | 0.0002 | 0.0007 | negative |
| * Technical NGS Cut-Off for specific variant, based on false-positive rate (mean+3*StDiv) | | | | | | | | | |
| ** ctDNA positive samples are shown in bold | | |  |  |  |  |  |  |  |


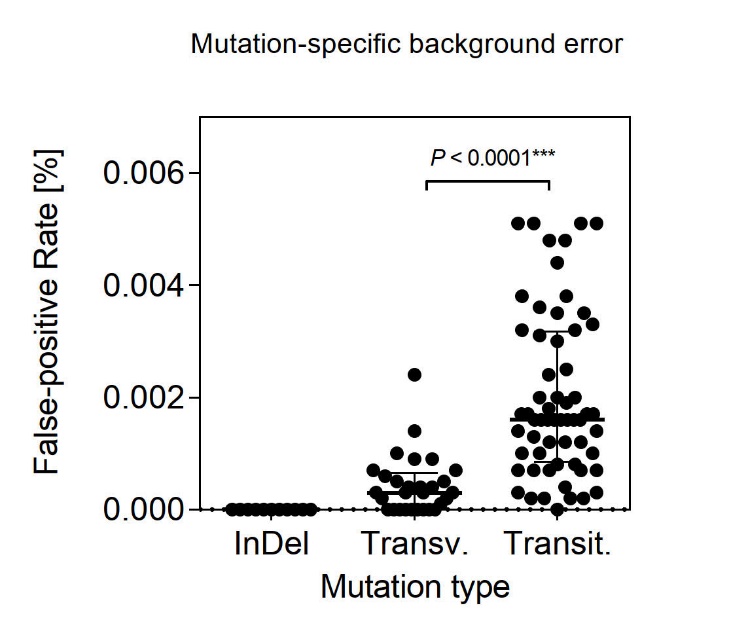


**Supplementary Figure 1.** Mutation type-specific false-positive rate (background error) for insertion/deletion (InDel) mutations, transition (C>T/T>C; A>G/G>A) and transversion (G>C/C>G; G>T/T>G; A>C/C>A; A>T/T>A) mutations.
